# What Causes the Discrepancy in SARS-CoV-2 Vaccine Between Parental Hesitancy for Themselves and for Their Children During Lockdown Period?

**DOI:** 10.1007/s44197-023-00122-3

**Published:** 2023-06-28

**Authors:** Tianshuo Zhao, Chao Wang, Sihui Zhang, Linyi Chen, Bingfeng Han, Hanyu Liu, Mingzhu Xie, Xianming Cai, Shanshan Zhang, Yiguo Zhou, Guoxing Li, Bei Liu, Juan Du, Jing Zeng, Yaqiong Liu, Qingbin Lu, Fuqiang Cui

**Affiliations:** 1 Department of Laboratorial Science and Technology, School of Public Health, Peking University, Beijing, 100191 People’s Republic of China; 2 Vaccine Research Center, School of Public Health, Peking University, Beijing, 100191 People’s Republic of China; 3 Puyang Center for Disease Control and Prevention, Henan, 457005 People’s Republic of China

**Keywords:** Vaccine hesitancy, SARS-CoV-2 vaccine, Parent, Health belief model, Structural equation model

## Abstract

**Background:**

Parents are usually the decision-makers for vaccinations of children. Therefore, it is important to understand parental beliefs and attitudes toward severe acute respiratory syndrome coronavirus 2 (SARS-CoV-2) vaccine for themselves and their children when it was approved for children age 3–17.

**Method:**

A cross-sectional survey based on an anonymous online questionnaire for parents was conducted in seven provinces of China, and demographic information, vaccination history, parental decision motives, and health belief model toward themselves and their children were collected, respectively.

**Results:**

The overall parental hesitancy rate toward themselves was 20.30%, and that toward their children was 7.80%. More parental concerns on disease severity (odd ratio [OR] = 1.11, 95% confidence interval [CI]: 1.01–1.61) and susceptibility (OR = 1.29, 95% CI: 1.01–1.63) of children could be the causes of discrepancy in hesitancy for themselves and for their children. Parents who hesitated to vaccinate themselves might also be hesitated to vaccinate their children (*β* = 0.077, *P* < 0.001).

**Conclusion:**

Threat perception may lead to inconsistencies in parental vaccination decisions toward themselves and toward their children. Correcting misinformation and strengthening education about COVID-19 are of great significance in addressing vaccine hesitancy among parents and children.

## Introduction

Coronavirus disease 2019 (COVID-19), caused by severe acute respiratory syndrome coronavirus 2 (SARS-CoV-2), has more than 517 million confirmed cases, including 6 million deaths globally. [1] Vaccines are one of the most successful non-drug interventions in the early stages, when specialized treatments that can be put out on a broad scale are lacking. It was estimated that vaccinations led to a global reduction of 63% in total deaths during the first year of SARS-CoV-2 vaccination. [2] The coverage rate of administering about 80% can sustain a reduction of confirmed cases and number of deaths [3]. As of May 16, 2022, 65.4% of the world’s population has received at least one dose of the SARS-CoV-2 vaccine. A total of 11.65 billion doses have been administered globally, including over 3.3 billion doses in China. [4] Vaccine scandal and hesitancy is more likely to emerge when vaccines are authorized for widespread use, affecting vaccination coverage in many countries. In China, SARS-CoV-2 vaccines have been approved for children aged 3–17 since June, 2021. [5] Vaccine hesitancy causes anxiety in parents, which may lead to refusal or delay to get their children vaccinated. For children and adolescents, parents are usually the decision-makers when it comes to vaccinations. Therefore, it is important to understand parents’ beliefs and attitudes toward SARS-CoV-2 vaccines both for them and their children. [6, 7] In accordance to studies, parents who are willing to vaccinate themselves against influenza, human papillomavirus, COVID-19 are more likely to vaccinate their children equally. [8–10] However, the lack of available evidence does not provide an accurate picture of the discrepancy in SARS-CoV-2 vaccine between parental hesitancy for themselves and for their children during lockdown period.

It is important to understand the factors influencing population vaccine hesitancy through mature survey tools or research theories on universal vaccination. [11] The health belief model (HBM) is one of the most widely used frameworks in the study of health behavior, including vaccination behavior. HBM primarily consists of four components: perceived threat, perceived benefit, perceived barrier, and self-efficacy. Other modules can be added for different research purposes, including self-efficacy and cues to action. The HBM has proven to be a simple and powerful theoretical framework for analyzing the determinants of vaccination behavior and willingness to vaccinate. Strong threat perception and benefit perception can promote people to vaccinate against COVID-19, while barrier perception, on the contrary, can hinder their administration. [12–14] Furthermore, HBM studies on SARS-CoV-2 vaccines have confirmed the applicability of this theory on vaccination willingness or hesitancy. [15, 16]

Therefore, this study investigated parental refused and delayed behaviors toward their children and themselves on SARS-CoV-2 vaccination in seven provinces in China. Combined with the vaccination decision survey and health belief model, we explored the influence factors of vaccine hesitancy and the inconsistencies in parental vaccination decisions for themselves and for their children, to provide scientific evidence for improving the coverage of SARS-CoV-2 vaccination among parents and children.

## Materials and Methods

### Sample and Data

In December 2021, an anonymous online questionnaire was developed and managed using Questionnaire Star (Changsha Ranxing Information Technology Co., Hunan, China), a platform providing functions including data collection, storage, and analysis. Seven provinces in China’s eastern, central, and western regions were selected as survey sites to promote the electronic questionnaire. Parents from Beijing and Shandong in eastern China, Heilongjiang, Henan, and Anhui in central China, and Gansu and Sichuan in western China were invited to fill out the online questionnaire through an internet communication platform. The researchers used WeChat, China's most widely used instant messaging service, to advertise recruitment and spread questionnaire links using snowball sampling. This study was approved by the ethics committee of the Peking University Health Science Center, China (approval number: IRB00001052-21132), and a signal-free informed consent application was approved. Before completing the online questionnaire, the participants were informed that their participation was voluntary and that their informed consent was obtained by submitting the questionnaire.

The inclusion criteria were Chinese adults aged 18 years and older, parents of children aged 3–17, informed consent, and voluntary participation.

### Measures of Variables

The questionnaire used in this study was divided into 4 parts with 32 items. But six items in the decision motives and nine items in the health model required parents to answer twice from their own perspective and from their children's perspective, respectively.

1. Vaccination Behavior and Willingness

**Fig. 1:**
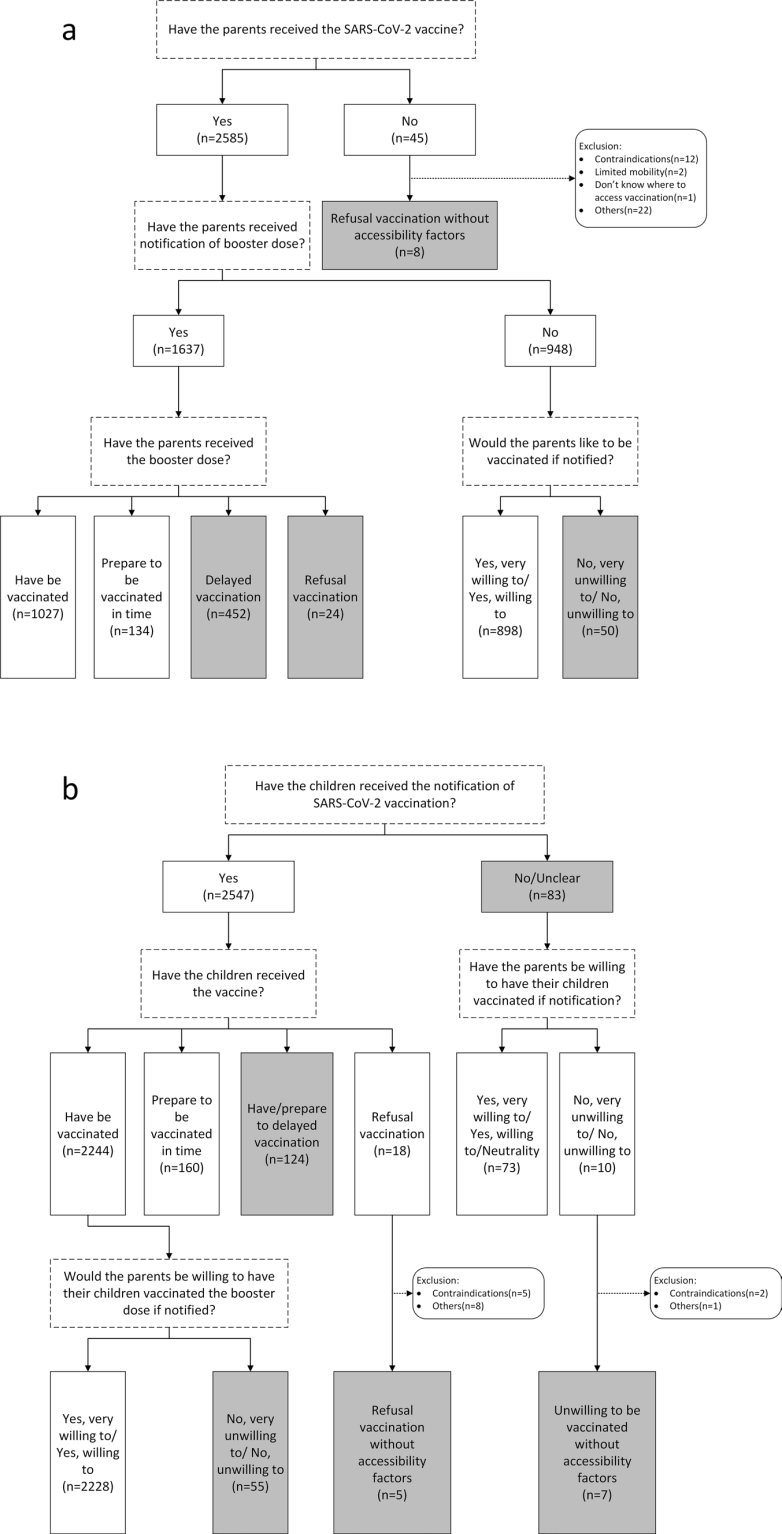
SARS-CoV-2 vaccination behaviors and willingness. The options in gray are defined as vaccine hesitancy. **a** The vaccination behaviors and willingness of parents; **b** the status of children and parental willingness toward children

The questionnaire collected ten items on participants’ SARS-CoV-2 vaccination behavior and willingness for themselves and for their children. Figure 1a shows the detailed inquiry process regarding parents’ vaccination status and Fig. 1b regarding vaccination status of children.

Parents were defined as having vaccine hesitancy toward themselves if one of the following conditions were met and vaccine availability factors were excluded (including parents having vaccine contraindications or mobility restrictions, not knowing where to get vaccinated, local vaccine shortages, etc.): ^①^ parents refused to get vaccinated against SARS-CoV-2; ^②^ parents have been notified of booster dose and have refused/delayed vaccination; and ^③^ parents did not receive the booster shot notification and were unwilling to receive the booster dose. (Fig. 1a).

Parents were defined as having vaccine hesitancy toward their children if one of the following conditions were met and vaccine availability factors were excluded (including children having vaccine contraindications or limited mobility, parents not knowing where to get their children vaccinated, local vaccine shortages, etc.): ^①^ children had been notified of SARS-CoV-2 vaccine, and parents refused/delayed their child's vaccination; ^②^ children did not receive vaccination notification and parents did not want to vaccinate their children if received; and ^③^ children have been vaccinated, but parents were reluctant to give their children a booster dose if health policies allow it in the future. (Fig. 1b).

We classified all parents into four vaccine hesitation groups based on their own and their children’s vaccine hesitancy. ^①^ P0C0: parents were not hesitant to vaccinate either themselves or their children; ^②^ P1C0: parents were only hesitant to vaccinate themselves; ^③^ P0C1: parents were only hesitant to vaccinate their children; and ^④^ P1C1: parents were hesitant to vaccinate both themselves and their children.

(2) Decision Motives of Vaccination

Six factors were included in the questionnaire that could affect parents’ decisions regarding the SARS-CoV-2 vaccine. They were listed as choices simultaneously in a multiple-response, including vaccine effectiveness, vaccine safety, disease severity, susceptibility to disease, reward/punishment (vaccination would be rewarded/non-vaccination would be punished), and group influence (vaccination behavior of family members and friends). Parents were informed that they should choose and rank the three most important factors leading to the vaccination decisions on themselves and on their children, respectively. According to the sorting order, the three selected factors were assigned three points, two points, and one point, respectively, and the factors not selected were assigned zero points.

(3) Health Belief Model

The scale was composed of perceived threat, perceived benefit, perceived barrier, and self-efficacy based on the HBM. It contained nine items in total, as presented in Supplemental Materials. ^①^ Perceived threat (perceived severity and perceived susceptibility) consists of three items, which could assess the perception of the possibility and consequences of SARS-CoV-2 infection. ^②^ Perceived benefit (two items) refers to parental evaluation of the effects brought by administrating SARS-CoV-2 vaccines, including the prevention on the onset, severity, and death of COVID-19. ^③^ Perceived barrier (two items) refers to the perception of obstacles encountered including the amount of effort and money spent on vaccination and the adverse effects of the vaccine. ^④^ Self-efficacy (two items) assesses the assurance in the decision to implement SARS-CoV-2 vaccination.

All items were scored 1–5 on a Likert scale (strongly disagree, disagree, neutral, agree, and strongly agree), with higher scores indicating higher perceptions. Parents were asked to answer the questions twice (for themselves and for their children) to investigate the health beliefs about themselves and their children. The additive scores of the items subordinate to each perception were calculated. And the Cronbach's alpha coefficient is used to determine the prerequisite that these items can be summed up.

(4) Social Demographic Information

Social demographic information included regions, parents’ sex, age, income, education, and children’s sex and age.

### Models and Data Analysis Procedure

Continuous variables were described as means and standard deviations, and classification variables were described in the form of frequency and percentage. Univariate tests between groups of categorical variables were performed using the Chi-square test or Fisher’s exact probability test, while rank variables were tested using the Kruskal–Wallis test.

A paired two-sided *t* test was used to check whether there was a difference between the score of the parent scale and the score of the child’s decision motivation. Logistic regression models were used to assess the dual differences in scores between the P0C1 and P1C0 groups and between parents and children, including sociodemographic variables as covariates. The results of the regression analysis were expressed as odds ratios (OR) and 95% confidence intervals (CI).

Structural equation modeling (SEM) with maximum likelihood estimation was constructed to examine the relationship between parental health beliefs and vaccine hesitancy toward themselves and children, respectively. Reliability evaluation and confirmatory factor analysis (CFA) were performed before modeling. Reliability was evaluated using Cronbach’s α, average variance extracted (AVE), and combination reliability (CR). In SEM, Cronbach’s α and CR > 0.7 is defined as good, and CR > 0.3 as acceptable; AVE > 0.5 is defined as good, and AVE > 0.3 as acceptable. CFA was used to conduct the measurement model to delineate the relationships between observed and latent variables and examine the interrelationships and covariation among observed variables. Factor loadings and the factor load of each observed variable should be over 0.3. Four indices were used to evaluate if the proposed model was supported: goodness of fit index (GFI), comparative fit index (CFI), Tucker–Lewis index (TLI), root mean square error of approximation (RMSEA), and standardized root mean squared residual (SRMR). The GFI, CFI, and TLI levels should be > 0.90, and RMSEA and SRMR should be < 0.08. When the fit indices were satisfactory, the path coefficients in the SEM were further scrutinized.

The results were considered statistically significant at *α* = 0.05. When multiple comparisons were performed, the corrected α_c_ = α/N was used for N-pairwise comparisons. Statistical analyses were performed using Stata 16.0 (Stata Corporation, College Station, TX, USA) and IBM SPSS Amos 26.0.0 (IBM Corporation, Armonk, NY, USA).

## Results

### Characteristics of Enrolled Participants Grouped by Vaccine Hesitancy Status

A total of 2,630 questionnaires were collected from parents with children aged 3–17, including 2,030 mothers and 600 fathers with an average age of 37 years. The vaccination coverage of parents receiving at least one dose of the SARS-CoV-2 vaccine was 98.29% (2,585/2,630).

Among 1,637 people who received notification of a booster dose, the booster coverage was 62.74% (1,027/1,637); among the 948 people who did not receive the booster vaccination notification, 94.72% (898/948) were willing to be vaccinated. Combined with the above status and excluding the availability factors of vaccination, the hesitancy rate among parents for SARS-CoV-2 vaccines was 20.30% (534/2,630). (Fig. 1a).

Among the children of these parents, 2,547 had been notified to receive the SARS-CoV-2 vaccine, 2,244 (88.10%) had received at least 1 dose of the SARS-CoV-2 vaccine, and most parents were also willing to let their children receive a booster dose in the future (99.29%, 2,228/2,244). Considering the above factors and excluding the availability of vaccines, 7.80% (191/2,630) of the parents hesitated to vaccinate their children against SARS-CoV-2. (Fig. 1b).

We classified all parents into four groups based on parental hesitancy toward their own and their children’s vaccine hesitancy, namely, non-hesitancy toward both parents and their child (P0C0 = 1972), hesitancy toward the parent only (P1C0 = 467), hesitancy toward their child only (P0C1 = 124), and hesitancy toward both parents and their child (P1C1 = 67). The basic demographic information of each group was described, respectively, as shown in Table 1. The Kruskal–Wallis test showed that there were differences in the distribution of age, education, monthly income, and age of children among the four groups. The Chi-squared test showed that there were differences in the distribution of regions among the four groups. Pairwise comparisons between P0C1, P1C0, and P1C1 and P0C0 were performed. The results showed that parents in the P0C1 and P1C0 groups were younger than those in the P0C0 group (*P* < 0.001). Parents who hesitated about their children, namely P0C1 and P1C1, had higher education and income levels than those of P0C0 (*P* < 0.001; *P* = 0.001). All parents with vaccine hesitancy, including P0C1, P1C0, and P1C1, had younger children than those in P0C0 (*P* < 0.001; *P *= 0.005; P < 0.001). In addition, parents of the P0C0 and P1C0 groups were mostly from the middle regions while those of the P0C1 and P1C1 groups were mostly from the eastern and western regions, respectively.

**Table 1:** The characteristics of enrolled subjects grouped by vaccine hesitancy status during lockdown period in China

Variable	Vaccine hesitancy status					*χ*^2^	*P* value
	P0C0 (*n* = 1972)	P1C0 (*n* = 467)	P0C1 (*n* = 124)	P1C1 (*n* = 67)	Total (*N* = 2630)		
Region^a^						10.181	0.017
East	369 (71.93)	80 (15.59)	45 (8.77)	19 (3.7)	513 (100)		
Middle	978 (77.93)	219 (17.45)	42 (3.35)	16 (1.27)	1255 (100)		
West	625 (72.51)	168 (19.49)	37 (4.29)	32 (3.71)	862 (100)		
Age of parents, *n* (%)^a^						31.184	< 0.001
< 30 years old	230 (11.66)	67 (14.35)	23 (18.55)	8 (11.94)	328 (100)		
30–35 years old	556 (28.19)	150 (32.12)	48 (38.71)	26 (38.81)	780 (100)		
35–40 years old	565 (28.65)	146 (31.26)	25 (20.16)	18 (26.87)	754 (100)		
More than 40 years old	621 (31.49)	104 (22.27)	28 (22.58)	15 (22.39)	768 (100)		
Sex of parents, *n* (%)^b^						1.012	0.798
Male	450 (22.82)	109 (23.34)	29 (23.39)	12 (17.91)	600 (100)		
Female	1522 (77.18)	358 (76.66)	95 (76.61)	55 (82.09)	2030 (100)		
Area of parents, *n* (%)^b^						7.726	0.052
Urban	1216 (61.66)	286 (61.24)	91 (73.39)	45 (67.16)	1638 (100)		
Rural	756 (38.34)	181 (38.76)	33 (26.61)	22 (32.84)	992 (100)		
Educational attainment of parents, *n* (%)^a^						51.109	< 0.001
Junior high school or below	948 (48.07)	221 (47.32)	34 (27.42)	19 (28.36)	1222 (100)		
Senior high school	396 (20.08)	113 (24.20)	19 (15.32)	16 (23.88)	544 (100)		
Bachelor degree or above	628 (31.85)	133 (28.48)	71 (57.26)	32 (47.76)	864 (100)		
Personal monthly income (RMB), *n* (%)^a^						33.220	< 0.001
< 2,000	689 (34.94)	171 (36.62)	26 (20.97)	17 (25.37)	903 (100)		
2,000–5,000	897 (45.49)	189 (40.47)	54 (43.55)	26 (38.81)	1166 (100)		
More than 5,000	386 (19.57)	107 (22.91)	44 (35.48)	24 (35.82)	561 (100)		
Age of children, *n* (%)^a^						78.804	< 0.001
< 7 years old (pre-school)	537 (68.76)	140 (17.93)	72 (9.22)	32 (4.10)	781 (100)		
7–12 years old (primary school)	682 (73.97)	193 (20.93)	27 (2.93)	20 (2.17)	922 (100)		
More than 12 years old (high school)	753 (81.23)	134 (14.46)	25 (2.70)	15 (1.62)	927 (100)		
Sex of children, n (%)^b^						5.966	0.113
Male	1109 (56.24)	283 (60.60)	61 (49.19)	37 (55.22)	1490 (100)		
Female	863 (43.76)	184 (39.40)	63 (50.81)	30 (44.78)	1140 (100)		

*P0C0* parents were not hesitant to vaccinate either themselves or their children, *P1C0* parents were only hesitant to vaccinate themselves, *P0C1*, parents were only hesitant to vaccinate their children, *P1C1* parents were hesitant to vaccinate both themselves and their children

^a^The statistics and P values were calculated by Pearson Chi-squared test

^b^The statistics and P values were calculated by Kruskal–Wallis test

### Parental Inconsistencies of Decision Motivations Toward Themselves and Children

**Fig. 2:**
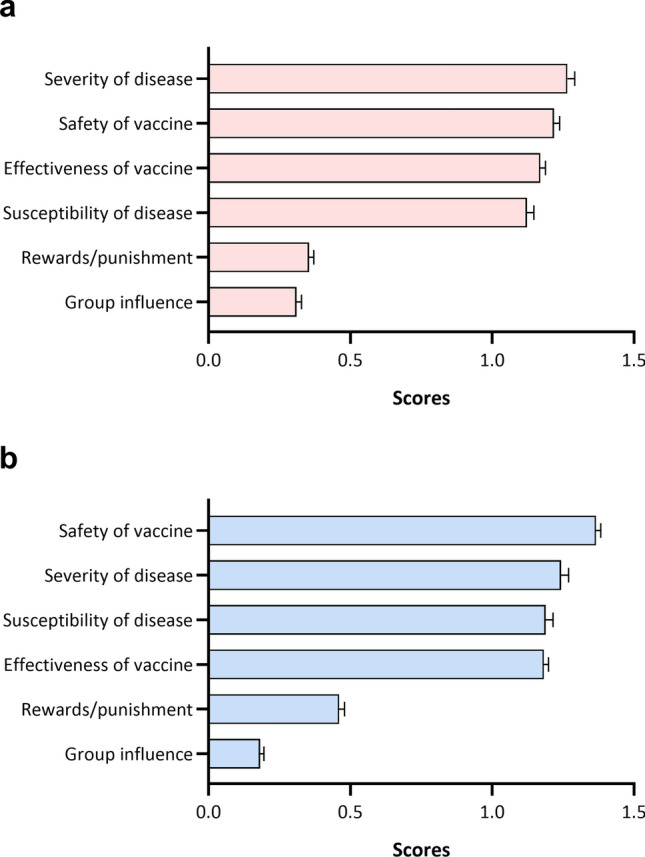
Decision motivation scores to accept or refuse SARS-CoV-2 vaccination. Three most important motivations were selected and ranked. And 3 points, 2 points, and 1 point assigned according to order, following with 0 points for non-selected ones. **a** Parental mean scores toward their own vaccination; **b** parental mean scores toward their children’s vaccination

We explored the parental decision motivations of toward themselves and children to accept or refuse vaccines. Scores were assigned according to the importance order of each motivation. For parents, the most important motivation influencing SARS-CoV-2 vaccination was disease severity (1.26 ± 0.03), followed by vaccine safety (1.22 ± 0.02) and effectiveness of the vaccine (1.17 ± 0.02), as shown in Fig. 2a. However, the most important factor influencing parents’ decision to vaccinate their children was vaccine safety (1.37 ± 0.02), followed by disease severity (1.24 ± 0.03) and susceptibility to disease (1.19 ± 0.03), as shown in Fig. 2b.

**Fig. 3:**
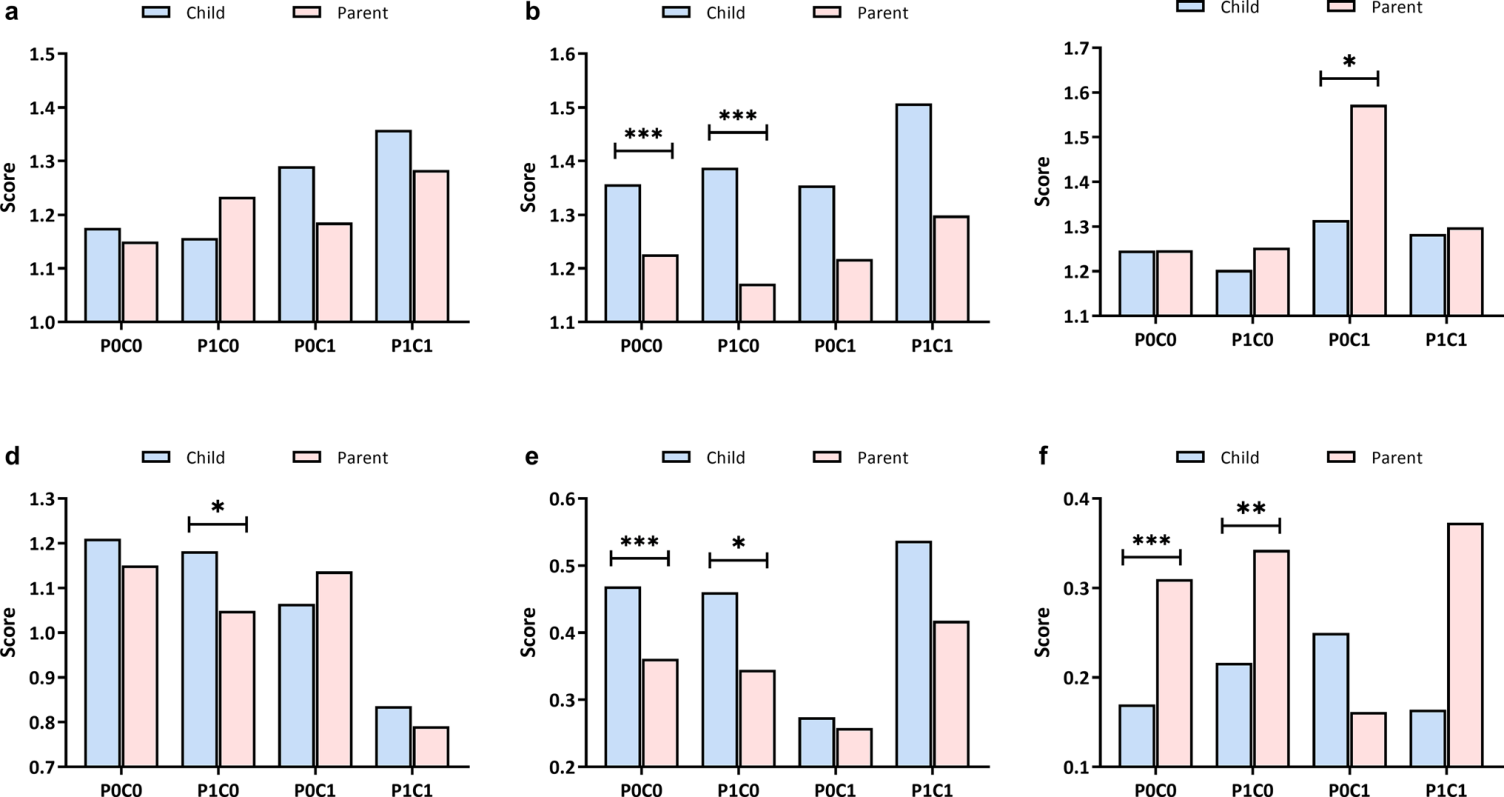
Intra-group decision motivation score comparison between parents and children. Within each group, the paired *t* test was used to compare differences in parental decision motivation scores for their own and their children’s vaccination (*P < 0.05, **P < 0.01, ***P < 0.001). **a** Effectiveness of vaccine; **b** safety of vaccine; (**c** severity of disease; **d** susceptibility of disease; **e** rewards/punishment; **f** group influence (vaccination behavior of family members and friends around). *P0C0* parents were not hesitant to vaccinate either themselves or their children, *P1C0* parents were only hesitant to vaccinate for themselves, *P0C1* parents were only hesitant to vaccinate for their children, *P1C1* parents were hesitant to vaccinate both themselves and their children

We used a paired *t* test to compare the score differences between the intragroup, as shown in Fig. 3. Except for the P1C0 group, parental vaccine effectiveness scores toward children were higher than those toward themselves, but the difference was not statistically significant (Fig. 3a). Parents in all groups had higher vaccine safety scores toward children than themselves, but only the P0C0 and P1C0 groups showed statistically significant differences (Fig. 3b). Parental disease severity score toward themselves in group P0C1 was higher than that toward the children, while there was no significant difference in the other groups (Fig. 3c). Parents in the P1C0 group had higher disease susceptibility scores toward children than themselves, while the P0C1 group had the opposite, but without statistical significance (Fig. 3d). Parents from all groups had higher reward and punishment scores toward children than toward themselves, but only the P0C0 and P1C0 groups had statistically significant differences (Fig. 3e). Parents of P0C0, P1C0, and P1C1 scored higher for themselves than for their children in the group influence factor, whereas the P0C1 group scored the opposite. The differences between the P0C0 and P1C0 groups were statistically significant. (Fig. 3f).

A logistic model was constructed to compare the score gap of parental motives toward themselves and children (i.e., the independent variables were the difference of the parental score for themselves minus for their children in each dimension) between the P1C0 and P0C1 groups. After adjusting for the sociodemographic characteristics of parents and children, we compared the dual differences between the two inconsistent groups, P1C0 and P0C1, as parental scores for themselves minus for their children, as shown in Table 2. Regression results showed that the dual difference in vaccine safety and disease severity between P0C0 and P1C0 had significant effect values. Compared with P1C0, the occurrence of P0C1 increased by 1.27 (95% CI 1.01–1.61) and 1.29 (95% CI 1.01–1.63) times for each 1-point gap increase (i.e., the score differences between parental scale for themselves and parental scale for children increase) in the two dimensions, vaccine safety and disease severity, respectively. This suggests that parents in the P1C0 group were more concerned about the severity and susceptibility of the disease in children than those in the P0C1 group, but less so in adults.

**Table 2:** The multivariate logistic regression model on the difference in parental vaccination decision scores toward themselves and toward their children between parents were only hesitant to vaccinate for themselves and parents were only hesitant to vaccinate for their children

Variable^a^	Diff [Parent–Child], mean (SD)		OR (95% CI)^b^	*P* value^b^
	P1C0	P0C1		
Effectiveness of vaccine	0.08 (0.05)	− 0.10 (0.07)	0.99 (0.76–1.29)	0.954
Safety of vaccine	− 0.22 (0.05)	− 0.14 (0.08)	1.11 (0.85–1.45)	0.436
Severity of disease	0.05 (0.06)	0.26 (0.12)	1.27 (1.01–1.61)	0.045
Susceptibility of disease	− 0.13 (0.07)	0.07 (0.12)	1.29 (1.01–1.63)	0.039
Rewards/punishment	− 0.12 (0.05)	− 0.02 (0.08)	1.24 (0.92–1.67)	0.163
Group influence	0.13 (0.04)	− 0.09 (0.08)	0.88 (0.64–1.23)	0.460

*P0C0* parents were not hesitant to vaccinate either themselves or their children, *P1C0* parents were only hesitant to vaccinate themselves, *P0C1* parents were only hesitant to vaccinate their children, *P1C1*, parents were hesitant to vaccinate both themselves and their children

^a^The dependent variable was the two groups with inconsistent vaccine hesitancy status between children and parents (P1C0 group: *Y* = 0; P0C1 group: *Y* = 1)

The independent variables were the scores difference between parent scale and children scale (X1 = differences in scores of the effectiveness of vaccine dimension; X2 = differences in scores of safety of vaccine; X3 = differences in scores of severity of disease; X4 = differences in scores of susceptibility of disease; X5 = differences in scores of rewards/punishment; X6 = differences in scores of group influence)

The covariates were the sociodemographic characteristics, including region, age, sex, area, educational attainment, and personal monthly income of parents, and age and sex of children

^b^OR and P value are from the multivariate analysis

### Structural Equation Model of Vaccine Hesitancy Based on Health Belief Model

**Fig. 4:**
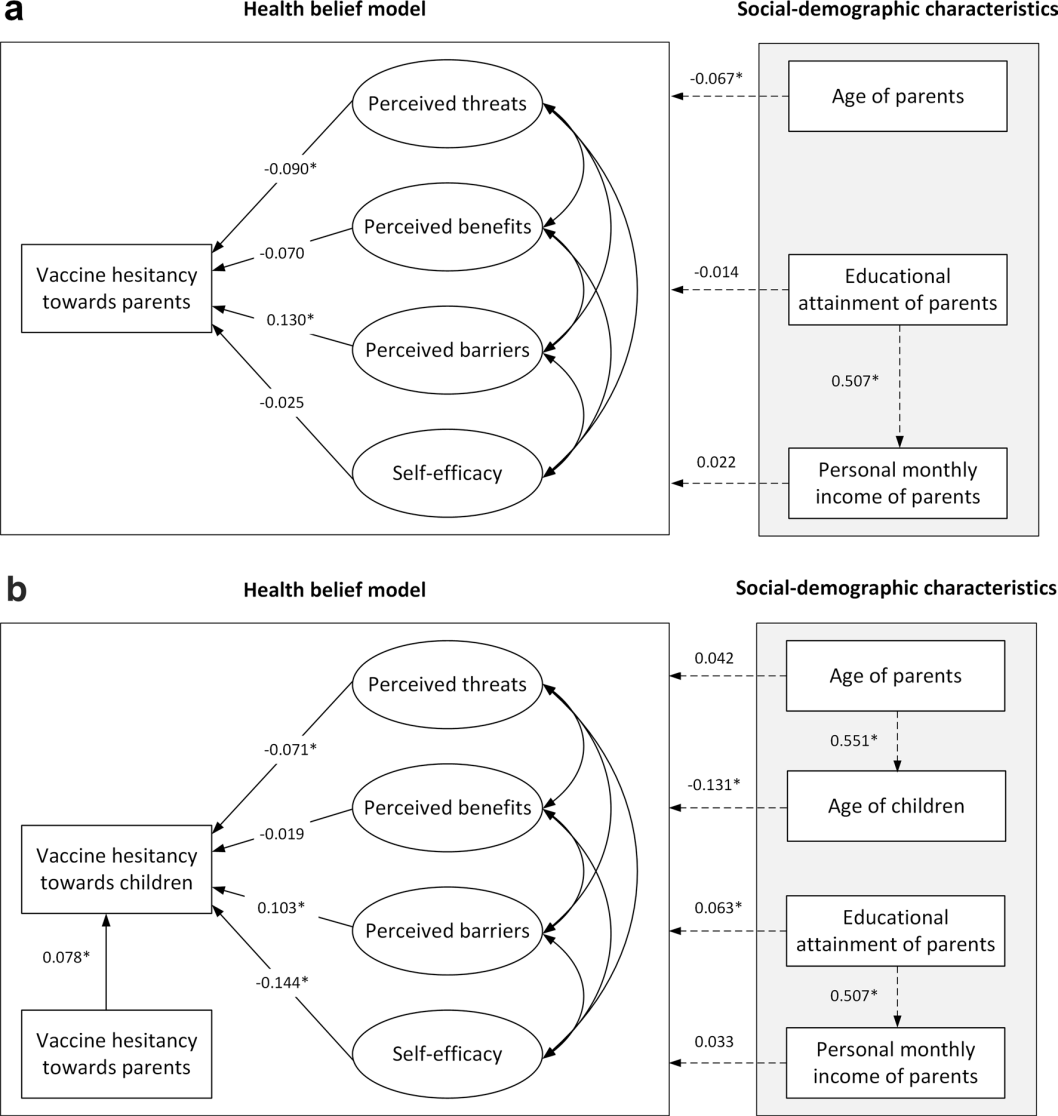
Structural equation model of vaccine hesitancy based on the health belief model. **a** Parental SARS-CoV-2 vaccine hesitancy toward themselves (*P < 0.05). **b**) Parental SARS-CoV-2 vaccine hesitancy toward their children (*P < 0.05)

The SEM of health beliefs toward parents and toward children was constructed according to the coefficient of each factor and the sociodemographic variables with inter-group differences. The preliminary fitting results showed that the factor load of item-Q3 was lower than 0.3. After removing this, the model was rebuilt (Fig. 4a, b).

Cronbach’s α values of perceived threat, perceived benefit, perceived barrier, and self-efficacy in the model were 0.714, 0.710, 0.548, and 0.461, respectively, within the acceptable reliability criteria of SEM construction. Other reliability results, including the AVE, CR, and Pearson correlation coefficients, are presented in Table 3. The factor loads of each item in the parent model ranged from 0.445 to 0.848, and those in the child model ranged from 0.437 to 0.857, both greater than 0.30, indicating that the model had good structural validity (Table 3). The fitting indices of the parent model were as follows: χ^2^ = 7.76, RMSEA = 0.05, SRMR = 0.06, GFI = 0.97, CFI = 0.93, and TLI = 0.90. The fitting index of the child model was χ^2^ = 8.13, RMSEA = 0.05, SRMR = 0.03, GFI = 0.97, CFI = 0.93, and TLI = 0.90, indicating that the fit of the model was acceptable.

**Table 3:** The reliability evaluation and factor load of health belief model

Variable	Cronbanch’ α	AVE	CR	Correlation coefficient	Factor loads
Parental vaccine hesitancy toward themselves					
Perceived threat	0.714	0.563	0.719	0.555	
Q1					0.686
Q2					0.809
Self-efficacy	0.461	0.426	0.578	0.360	
Q4					0.848
Q5					0.653
Perceived benefit	0.710	0.573	0.725	0.553	
Q6					0.445
Q7					0.809
Perceived barrier	0.548	0.388	0.559	0.387	
Q8					0.639
Q9					0.606
Parental vaccine hesitancy toward children					
Perceived threat	0.707	0.562	0.717	0.546	
Q1					0.658
Q2					0.831
Self-efficacy	0.459	0.428	0.579	0.357	
Q4					0.857
Q5					0.646
Perceived benefit	0.711	0.576	0.727	0.554	
Q6					0.437
Q7					0.816
Perceived barrier	0.542	0.381	0.552	0.381	
Q8					0.614
Q9					0.621

*AVE* average variance extract, *CR* combination reliability

In the model constructed based on parental health beliefs toward themselves in Fig. 3a, there was a positive impact on vaccine hesitancy from perceived barriers (*β* = 0.130, *P* < 0.001) and a negative impact from perceived threats (*β* = − 0.090, *P* = 0.002). The results of the abovementioned model corrected the influence of sociodemographic information on vaccine hesitation, including parental age (β = − 0.067, *P* < 0.001).

In the model constructed based on parental health beliefs toward their children, parents who hesitated to vaccinate themselves were more likely to have vaccine hesitancy behaviors or tendencies toward their children (*β* = 0.078, *P *< 0.001). Similar to the parental model, parental perceived barriers to their children had a positive effect on their hesitancy toward their children (*β* = 0.103, *P* = 0.003). On the other hand, perceived threats had a negative effect (*β* = − 0.071, *P* = 0.013). In addition, vaccine hesitancy was negatively affected by parental self-efficacy regarding their children's vaccination decisions (*β* = − 0.144, *P* = 0.001). The above model results corrected for the effects of sociodemographic information on vaccine hesitancy, including the age of the child (*β* = − 0.131, *P* < 0.001) and educational attainment of parents (*β* = 0.063, *P* = 0.001).

## Discussion

By the end of 2021, nationwide vaccination of adults with inactivated SARS-CoV-2 vaccine was completed in China, and booster dose vaccination was in progress. [17] The government first notified the public in June 2021 that the vaccine could be used in children aged 3–17 and encouraged parents to vaccinate their children against SARS-CoV-2. [5] Therefore, this study was designed under the background that vaccination for adults had almost been completed and that booster vaccination for adults and the first vaccination for children had been started voluntarily nationwide. Although the vaccination was organized by the health authority and was provided for free, people volunteered for the SARS-CoV-2 vaccines, and it was the parents’ decision to vaccinate the children. Through the investigation, the main results of our study were: ^①^ The overall parental hesitancy rate toward themselves (20.30%) was higher than toward their children (7.80%); ^②^ partial parental vaccination decisions toward themselves were inconsistent with those toward children (22.47%), which is due to parental perceived threat of COVID-19. That is, if parents feel that COVID-19 poses a greater threat to children than to adults, they would tend to vaccinate their children rather than themselves, and vice versa. ^③^ Parental vaccine hesitancy for themselves and for their children was positively correlated (*β* = 0.078). Through this study, the research gap was supplemented with the clarification on the inconsistency between parental hesitation for themselves and for children. And SEM was used to analyze the consistent correlation between the two instead of regression model.

### The Factors Influencing Parental Vaccine Hesitancy are Varied

Parents were worried about vaccinating their children. [18] A pooled result found that approximately 56.8% of parents intended to vaccinate their children against SARS-CoV-2, and this proportion varied greatly between societies. [19] However, in this study, 88.1% of the parents vaccinated their children after receiving the SARS-CoV-2 vaccine notification, which may be a result of the mobilization and advocacy of governments. In addition, this may be due to differences in the scope of vaccine hesitancy and willingness, as well as the differences between the willingness in the earlier period and the actual behavior in the later period. [20]

In this study, disease threat and vaccine safety were still significant factors that influenced parents’ vaccination decisions. Most parents care more about the safety and effectiveness of vaccines for their children than for themselves. The SEM results also showed that parental hesitancy toward themselves and their children was negatively affected by perceived threats of disease and positively affected by perceived barriers to vaccination. This result is supported by several previous study results. [15, 16, 22, 23] Vaccine hesitancy toward children was also influenced by parental self-efficacy, which suggests that parents may not have sufficient confidence and self-efficacy to vaccinate their children against SARS-CoV-2 due to knowledge reserves and information access. [26] Self-efficacy could lead to adjustments to overcome obstacles and risks, indirectly increasing vaccination willingness and reducing the risk of vaccine hesitancy. [26–28]

We also found that parental vaccine hesitancy was related to age, region, and educational level. Younger parents or those with younger children were more likely to have vaccine hesitancy, consistent with previous research [19, 29]. However, the effect of education on vaccine hesitancy is complex and controversial. Some studies have shown that low levels of education and cognition lead to increased concerns about vaccine efficacy, whereas others have shown the opposite, which may be related to time, region, society, and other factors. [30–34] Finally, vaccine hesitancy varied among regions. Parents in eastern China, where the economy is most developed, were more likely to hesitate to vaccinate their children. The relationship between economic and vaccine inclination is ambiguous. People in high-income region may weigh the pros and cons of vaccine safety and efficacy more because of high educational attainment and improved vaccine knowledge [35]. And the time and effort cost of vaccination for high-income parents is often higher than that for low-income parents, further increasing the precepted barrier [36]. Moreover, those in middle-to-high-income areas may have easier access to information through the internet and social media, which may lead to increased exposure to anti-vaccine information. [37]

### There are Consistency and Inconsistency in Parental Vaccine Hesitancy Toward Themselves and Their Children Simultaneously

Few studies have compared parental vaccine hesitancy toward themselves and their children. Since SARS-CoV-2 vaccines have been approved for people aged 3 years and older, evaluating the associations and inconsistencies between parents and children is appropriate. In this study, 22.5% of parents were inconsistent about their children’s and their own vaccine hesitancy, and the reasons for this lie in the different perceptions of disease threat between adults and children. Some parents were hesitant toward themselves because they believed that the threat of SARS-CoV-2 was greater for children. Some parents were hesitant toward their children for the opposite reason, related to the epidemiological evidence from the beginning of the outbreak, in which the infection was mostly mild, and children were not susceptible. [38] However, with the development of the epidemic and the evolution of the virus, several clustered outbreaks have been reported in kindergartens and primary schools, causing moderate and severe cases. [39] Therefore, incorrect information and threat perception mislead parents’ judgment. [40, 41] In addition, parents may hesitate to vaccinate their children by focusing excessively on group actions around them, such as the vaccination behaviors of family and friends toward their children. This also suggests that the behavior and tendency of vaccine hesitancy was communal aggregation, with the consciousness and behavior of a small group spilling over into the surrounding groups. [42, 43]

Although this study found some inconsistency between parents and children regarding vaccine hesitancy, there was a positive unidirectional association between parental hesitancy toward their children [44]. As decision-makers of children’s vaccination, the ideology and behavior of parents were directly projected on the health behavior of their children. [27] Parental positive attitudes on vaccines can influence their decision to vaccinate their children against SARS-CoV-2. For example, parents whose children have recently received flu vaccines or have a complete vaccination history reported that their children were more likely to receive SARS-CoV-2 and other vaccines. [19] This confirmed the relationship of vaccine hesitancy between parents and children.

### The Settlement to Parental Vaccine Hesitancy Requires a Multi-sectoral Effort

From a micro-perspective, we should lessen the cost of vaccination, especially vaccine safety, which suggest that governments should make vaccine information public in a timely manner to ensure that the current information is accurate, transparent, and scientific. SARS-CoV-2 vaccine counseling services and education campaigns provided by healthcare workers, who are influential actors in vaccination decisions, may be more effective in reducing parental concerns about the safety of SARS-CoV-2 vaccines. [19] The time and effort spent by parents in administering themselves and their children can also be considered as an indirect cost, and the location of vaccination sites may affect their vaccination behavior and willingness [24, 25]. Organizing group vaccination events regularly in residential areas, workplaces, and schools, hence, may help alleviate the impact of this issue. Beyond it, the distribution of vaccine hesitancy across sociodemographic characteristics suggests that young parents (or with young children) in economically developed regions are a key target for health education. The complex influence of education on parents also inspires that it is necessary to develop targeted publicity materials for highly educated parents rather than blindly encouraging and mobilizing them. It is important to provide scientifically accurate risk–benefit information to the public to improve vaccine coverage. The government has made vaccine information public in a timely manner to ensure the accuracy, transparency, and scientific nature of information currently in circulation. [19]

While from a macro-perspective, the positive effects of good governance (i.e., voice and accountability, government effectiveness, regulatory quality) is supporting the prompt administering of vaccinations and vaccine hesitancy [45]. Good governance also leads to high levels of vaccination through increased investment in vaccine research and development, vaccination campaigns, etc.

### Research Limitations

This study has many limitations. First, there would be self-report bias in this study, as some questions were based on the recall of the subjects. And the questionnaire was filled out voluntarily by the subjects interested in this study after published on the Internet, so response bias would be existed. Second, adults were vaccinated earlier than children were for health policy reasons. In the course of this study, the booster dose in adults and first dose in children were being gradually promoted across China. Therefore, for the booster dose, we could obtain coverage and willingness in adults, but only willingness in children. Vaccination intentions would change over time and do not necessarily translate into actual vaccination behavior in the future, which may contribute to the low rate of vaccine hesitancy in children and the high rate in adults. [20] Third, this study was conducted before the prevalence of Omicron variants in China and may not represent the current hesitancy status of parents and children. Third, we examined vaccine hesitancy using an online questionnaire; parents without a smartphone were unable to participate in our study. Although they account for a small proportion, they still impact the sample representation. Finally, the reliability results of the HBM items used to construct the SEM were acceptable and did not reach an optimal evaluation standard.

## Conclusion

In conclusion, parents were more likely to hesitate vaccinating themselves against COVID-19 than vaccinate their children. And there is inconsistency in parental vaccination decisions for themselves and for their children simulatively, which could be addressed by redressing the perceived threat of COVID-19. From a micro-perspective for lessening vaccine hesitancy, making vaccine information public in a timely manner to ensure the accurate, transparent, and scientific information has positive impact in correcting misinformation about vaccines and diseases. And population-specific health education and vaccination campaigns can improve vaccine hesitancy. From a macro-perspective, good governance and large financial input can promote the construction of public health system and vaccination.

## Data Availability

All data and statistical code to reproduce the results in the manuscript are available from the corresponding author upon reasonable request.

## Abbreviations

AVE: Average variance extracted
CFA: Confirmatory factor analysis
CFI: Comparative fit index
CI: Confidence interval
COVID-19: Coronavirus disease 2019
CR: Combination reliability
GFI: Goodness of fit index
HBM: Health belief model
OR: Odds ratio
RMSEA: Root mean square error of approximation
SARS-CoV-2: Severe acute respiratory syndrome coronavirus 2
SEM: Structural equation modeling
SRMR: Standardized root mean squared residual
TLI: Tucker–Lewis index

## References

[1] WHO. Coronavirus disease (COVID-2019) situation reports. 2021. https://www.who.int/emergencies/diseases/novel-coronavirus-2019 (Accessed Feb 19 2021).

[2] Watson OJ, Barnsley G, Toor J, Hogan AB, Winskill P, Ghani AC (2022). Global impact of the first year of COVID-19 vaccination: a mathematical modelling study. Lancet Infect Dis.

[3] Coccia M (2022). Optimal levels of vaccination to reduce COVID-19 infected individuals and deaths: a global analysis. Environ Res.

[4] Our World in Data. Coronavirus (COVID-19) Vaccinations. 2022. https://ourworldindata.org/covid-vaccinations.

[5] Joint Prevention and Control Mechanism of the State Council. The SARS-COV-2 vaccine can be used as an emergency for children aged 3–17. 2021. http://www.gov.cn/xinwen/2021-06/12/content_5617313.htm (Accessed Mar 27 2022).

[6] Brown C, Morlock A, Blakolmer K, Heidari E, Morlock R (2022). COVID-19 vaccination and race - a nationwide survey of vaccination status, intentions, and trust in the US general population. J Manag Care Spec Pharm.

[7] Ruiz JB, Bell RA (2022). Parental COVID-19 vaccine hesitancy in the United States. Public Health Rep.

[8] Kornides M, Head KJ, Feemster K, Zimet GD, Panozzo CA (2019). Associations between HPV vaccination among women and their 11–14-year-old children. Hum Vaccin Immunother.

[9] Kaufmann J, DeVoe JE, Angier H, Moreno L, Cahen V, Marino M (2022). Association of parent influenza vaccination and early childhood vaccinations using linked electronic health record data. Vaccine.

[10] Zhou Y, Li GX, Zhao TS (2023). Parents' willingness to vaccinate themselves and their children with the booster vaccine against SARS-CoV-2: A cross-sectional study in Puyang city, China. J Med Virol.

[11] WHO. The Guide to Tailoring Immunization Programs: Increasing coverage of infant and child vaccination in the WHO European Region. 2020. http://www.euro.who.int/__data/assets/pdf_file/0003/187347/The-Guide-to-Tailoring-Immunization-Programmes-TIP.pdf (Accessed June 3 2020).

[12] Brewer NT, Chapman GB, Gibbons FX, Gerrard M, McCaul KD, Weinstein ND (2007). Meta-analysis of the relationship between risk perception and health behavior: the example of vaccination. Health Psy Off J Division Health Psychol, Am Psychol Assoc.

[13] Mercadante AR, Law AV (2021). Will they, or Won’t they? Examining patients’ vaccine intention for flu and COVID-19 using the health belief model. Res Social Adm Pharm.

[14] Hall CM, Northam H, Webster A, Strickland K. Determinants of seasonal influenza vaccination hesitancy among healthcare personnel: An integrative review. J Clin Nurs 2021. 10.1111/jocn.16103 34716635

[15] Lin Y, Hu Z, Zhao Q, Alias H, Danaee M, Wong LP (2020). Understanding COVID-19 vaccine demand and hesitancy: a nationwide online survey in China. PLoS Negl Trop Dis.

[16] Chen H, Li X, Gao J (2021). Health belief model perspective on the control of COVID-19 vaccine hesitancy and the promotion of vaccination in China: web-based cross-sectional study. J Med Internet Res.

[17] National Health Commission. COVID-19 Diagnosis and Treatment Protocol (Version 9). 2022. http://www.nhc.gov.cn/yzygj/s7653p/202203/b74ade1ba4494583805a3d2e40093d88/files/ef09aa4070244620b010951b088b8a27.pdf.

[18] Bianco A, Mascaro V, Zucco R, Pavia M (2019). Parent perspectives on childhood vaccination: How to deal with vaccine hesitancy and refusal?. Vaccine.

[19] Galanis P, Vraka I, Siskou O, Konstantakopoulou O, Katsiroumpa A, Kaitelidou D (2021). Willingness and influential factors of parents to vaccinate their children against the COVID-19: a systematic review and meta-analysis. MedRxiv Preprint Server Health Sci.

[20] Byrne T, Patel P, Shrotri M (2021). Trends, patterns and psychological influences on COVID-19 vaccination intention: Findings from a large prospective community cohort study in England and Wales (Virus Watch). Vaccine.

[21] Li J-B, Lau EYH, Chan DKC (2022). Why do Hong Kong parents have low intention to vaccinate their children against COVID-19? testing health belief model and theory of planned behavior in a large-scale survey. Vaccine.

[22] Shmueli L (2021). Predicting intention to receive COVID-19 vaccine among the general population using the health belief model and the theory of planned behavior model. BMC Public Health.

[23] Mahmud I, Kabir R, Rahman MA, Alradie-Mohamed A, Vinnakota D, Al-Mohaimeed A (2021). The health belief model predicts intention to receive the COVID-19 vaccine in Saudi Arabia: results from a cross-sectional survey. Vaccines.

[24] MacDonald NE (2015). Vaccine hesitancy: definition, scope and determinants. Vaccine.

[25] Zhang Q, Shi Y, English AS (2022). COVID-19 Vaccine uptake in the context of the first delta outbreak in China during the early summer of 2021: the role of geographical distance and vaccine talk. Risk Manag Healthc Policy.

[26] Wang Y, Zhang X (2021). Influence of parental psychological flexibility on pediatric COVID-19 vaccine hesitancy: mediating role of self-efficacy and coping style. Front Psychol.

[27] Newman PA, Logie CH, Lacombe-Duncan A (2018). Parents’ uptake of human papillomavirus vaccines for their children: a systematic review and meta-analysis of observational studies. BMJ Open.

[28] Stout ME, Christy SM, Winger JG, Vadaparampil ST, Mosher CE (2020). Self-efficacy and HPV vaccine attitudes mediate the relationship between social norms and intentions to receive the hpv vaccine among college students. J Community Health.

[29] Fadzilatul AI, Leelavathi M, Petrick P (2023). Parental hesitancy and perception of the COVID-19 vaccine for children below 5 years in Cheras district. Kuala Lumpur Med J Malaysia.

[30] Larson H, Figueiredo A, Xiahong Z (2016). The state of vaccine confidence 2016: global insights through a 67-country survey. EBioMedicine.

[31] Weiss C, Schropfer D, Merten S (2016). Parental attitudes towards measles vaccination in the canton of Aargau, Switzerland: a latent class analysis. BMC Infect Dis.

[32] Justwan F, Baumgaertner B, Carlisle J, Carson E, Kizer J (2019). The effect of trust and proximity on vaccine propensity. PLoS ONE.

[33] Rosso A, Massimi A, De Vito C (2019). Knowledge and attitudes on pediatric vaccinations and intention to vaccinate in a sample of pregnant women from the City of Rome. Vaccine.

[34] Black S (2016). Recognizing the importance of vaccine confidence. EBioMedicine.

[35] Hudson A, Montelpare WJ (2021). Predictors of vaccine hesitancy: implications for COVID-19 Public health messaging. Int J Environ Res Public Health.

[36] Liu B, Cao B, Wang C (2023). Cost-minimization analysis of DTaP-IPV-Hib combination vaccine in China: a nationwide cross-sectional study. J Med Virol.

[37] Kennedy J (2020). Vaccine hesitancy: a growing concern. Paediatr Drugs.

[38] Rothan HA, Byrareddy SN (2020). The epidemiology and pathogenesis of coronavirus disease (COVID-19) outbreak. J Autoimmun.

[39] Information Office of Bejing Municipality. The 292nd press conference on COVID-19 prevention and control in Bejing. 2022. https://www.beijing.gov.cn/shipin/Interviewlive/645.html

[40] Chen B, Zhang J, Jiang Z, Shao J (2015). Media and public reactions toward vaccination during the ‘hepatitis B vaccine crisis’ in China. Vaccine.

[41] Larson H, Smith D, Paterson P, Cumming M (2013). Measuring vaccine confidence: analysis of data obtained by a media surveillance system used to analyse public concerns about vaccines. Lancet Infect Dis.

[42] Delamater P, Pingali S, Buttenheim A, Salmon D, Klein N, Omer S (2019). Elimination of nonmedical immunization exemptions in California and school-entry vaccine status. Pediatrics.

[43] Callender D (2016). Vaccine hesitancy: more than a movement. Hum Vaccin Immunother.

[44] Deng JS, Chen JY, Lin XQ, Huang CL, Tung TH, Zhu JS (2023). Parental hesitancy against COVID-19 vaccination for children and associated factors in Taiwan. BMC Public Health.

[45] Benati I, Coccia M (2022). Global analysis of timely COVID-19 vaccinations: improving governance to reinforce response policies for pandemic crises. Inter J Health Gover.

